# Supplementary material for: Impact of Neurons on Patient-Derived Cardiomyocytes Using Organ-On-A-Chip and iPSC Biotechnologies
Source: Cells. 2022 Nov 25;11(23):3764. doi: 10.3390/cells11233764 (PMC9737466; doi:10.3390/cells11233764)
Supplement: Supplementary file 1 [file cells-11-03764-s001.zip › cells-2007718-supplementary.pdf]

Supplemental Table S1: Mass spectrometry elution gradient.

**HPLC gradient**							
**Time (min)**	0	2.5	9	11	12	12.5	18
**% B mobile phase**	0	0	20	98	98	0	0

Supplemental Table S2: MS conditions.

**MS parameters**	
**Mode**	positive
**Spray voltage**	3,500 V
**Nebulizer gas**	Nitrogen
**Desolvation (nitrogen) sheath gas**	18 Arb
**Aux gas**	7 Arb
**Ion transfer tube temperature**	297°C
**Vaporizer temperature**	131°C
**Q1 and Q3 resolutions**	0.7 FWHM
**Collision gas (CID, argon) pressure**	2 mTorr

Supplemental Table S3: MS ionization, selection, fragmentation and identification parameters

**Compounds**	**Polarity**	**Precursor (m/z)**	**Product (m/z)**	**Collision Energy (V)**	**RF Lens (V)**
**Dopamine**	positive	324.14	116.11<br>145.11<br>171.05	54.24<br>33.36<br>23.90	206
**D4-Dopamine**	positive	328.14	116.11<br>128.05<br>171	55.00<br>48.67<br>20.76	206

**Adrenalin**	positive	354.11	166.04<br>171.06<br>184.04	18.09<br>27.69<br>14.60	179
**D6-Adrenaline**	positive	360.17	171.06<br>172.04<br>190.04	27.19<br>18.65<br>14.35	184
**Noradrenalin**	positive	340.15	116.11<br>145.11<br>171.06	55.00<br>36.19<br>24.61	215
**C6-Noradrenaline**	positive	346.15	145.11<br>158.11<br>171.06	36.04<br>18.34<br>25.62	226

Supplemental Table S4: Listing of antibodies used

	Species	*Epitope*	*Concentration*	*Reference*	*Supplier*
**Cardiac troponin I**	*Mouse*	IgG2b	1/500	4T21	Hytest
**alpha actinin**	*Mouse*	IgG1	1/1000	A7732	Sigma
**Beta3 tubulin**	*Mouse*	IgG2b	1/1000	T8660	Sigma
**Tyrosine Hydroxylase**	*Rabbit*		1/500	AB152	Merck
**Synapsin 1**	*Rabbit*		1/200	ab64581	Abcam
**ChAT**	*Mouse*	IgG2b	1/500	Mab_31384	Invitrogen
**DBH**	*Rabbit*		1/200	22806	Immunostar Inc.

Supplemental Figures:

Supplemental figure S1.

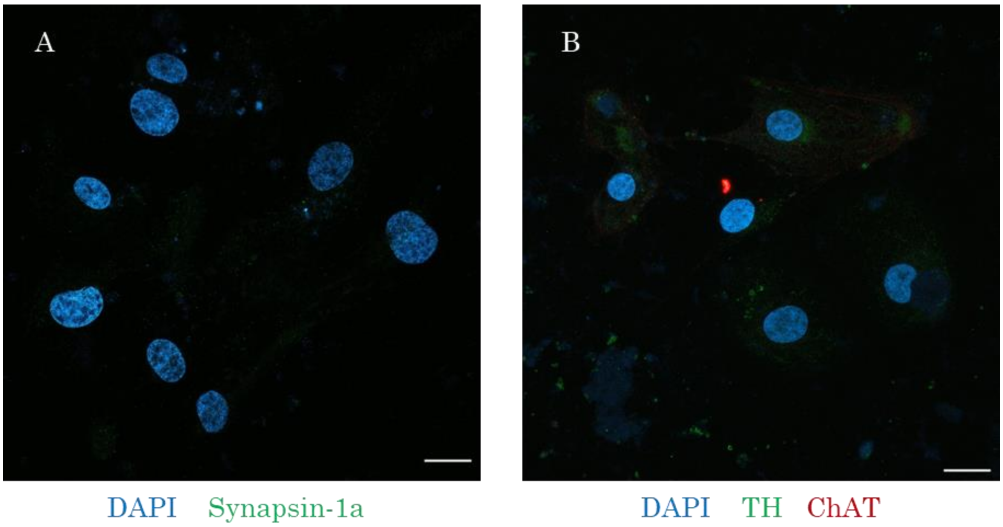

Supplemental figure S1: hiPSC-CMs alone in OOC do not express synapsin-1a and tyrosine hydroxylase.

Immunocytochemistry of 30-days-old hiPSC-CMs cultivated alone in OOC. DNA (DAPI) is stained in blue with (A) synapsin-1a in green or (B) tyrosine hydroxylase in green and choline acetyltransferase in red. Scale bar: 20 µm

Supplemental figure S2.

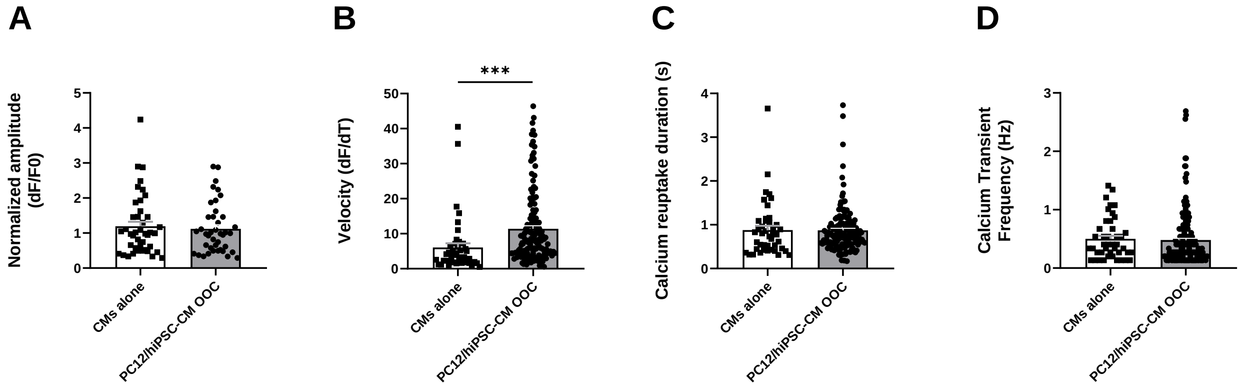

Supplemental Figure S2: SR calcium handling properties in hiPSC-CMs with or without PC12.

Monitoring of the intracellular calcium cycling properties in hiPSC-CMs cultivated on microfluidic devices alone or in presence of PC12 for 14 days. (A) Normalized amplitude of CaT, (B) calcium release velocity, (C) calcium reuptake duration and (D) CaT frequency. Mann Witney test, ***, $p<0.001$.

Supplemental figure 3.

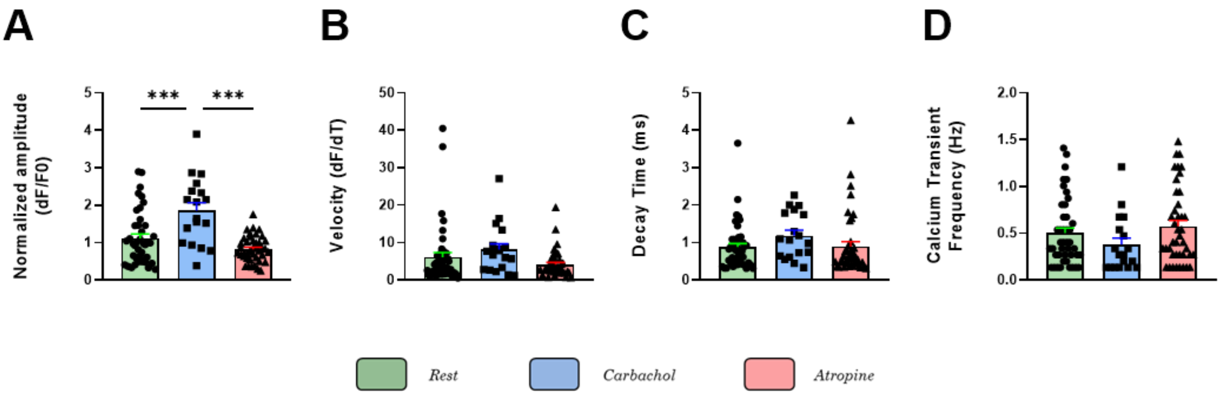

Supplemental figure 3 : Effect of carbachol and atropine on SR calcium handling of hiPSC-CMs cultivated in absence of PC12 in microfluidic devices.

Carbachol (1.5 mM) were applied in hiPSC-CMs while atropine (1µM) was applied in neuronal compartment in absence of PC12. (A) Normalized amplitude, (B) calcium release velocity, (C) calcium reuptake duration and (D) CaT frequency. The hiPSC-CMs were cultivated alone on microfluidic devices for 14 days. Tukey's multiple comparisons test, ***, $p<0.001$.
